# Visual Field Loss: Integrating Overlayed Information to Increase the Effective Field of View

**DOI:** 10.3390/vision6040067

**Published:** 2022-11-11

**Authors:** Jordi M. Asher, Paul B. Hibbard

**Affiliations:** Department of Psychology, University of Essex, Colchester CO4 3SQ, UK

**Keywords:** visual field loss, scotoma, hemianopia, augmented reality, assistive technology, rehabilitation

## Abstract

Visual field loss is a debilitating impairment that can impact normal daily activities. The advancement of augmented and virtual realities brings opportunities for potential substitutive technologies for visual field loss. Here we outline a conceptual approach to increasing the amount of useful information by overlaying the blind field into the sighted field. In this proof-of-concept experiment, 33 observers were allocated to either a left or right blind condition (with a simulated scotoma). All observers completed a line bisection task in all three conditions (baseline, scotoma, manipulation), with the baseline condition always completed first. The scotoma condition (baseline with the addition of a simulated scotoma) and the manipulated condition (baseline with the addition of a simulated scotoma, and a “minified window overlay”) were randomised in order of presentation. Predictably, our results show that a simulated scotoma impaired performance on the task. However, observers were able to make use the overlay to improve their estimation of the line’s midpoint. Our results show that a substitutive augmentation of this type improved accuracy in estimating the midpoint of a line with a (simulated) scotoma.

## 1. Introduction

Visual defects are one of the most common and debilitating consequences of brain injuries such as stroke and brain injury, estimated to affect between 20% and 57% of survivors [1,2]. These figures are thought to be under-reported, such that the prevalence is in fact likely to be higher than this estimate [3]. Visual impairments can include deficits in central or peripheral vision such as eye movement deficits [4] and perceptual problems including reduced acuity [5], visual field defects [6], and visual neglect [7]. Visual field loss can occur when the visual pathway has been damaged by a cortical stroke [8] or other brain injury.

The range of the visual field in the average human measures approximately 90${}^{\circ }$ to the left and right, 50${}^{\circ }$ above and 60${}^{\circ }$ below central vision [9]. Blind fields (scotomas) can affect either central or peripheral visual fields, however for the purpose of this research we generally focus on the near and far periphery rather than central vision. The loss of information in peripheral vision is significant since it plays an important role in evaluating the gist of an environment [10], selecting regions (through exogenous attention) for foveation and scrutiny [11] and even for reading [12].

People with visual field loss can experience severe impacts on their functional ability and report a reduced quality of life [5]. The impact of sight loss, whether from stroke or other causes, can be extremely disruptive to everyday life, severely impairing reading, mobility, independence, and ability to participate in rehabilitation, which can result in social isolation, and create depression and anxiety [13,14].

### 1.1. Restitution, Compensation, Substitution

In contrast to the intensive therapies available for stroke patients with damage to the motor cortex, cortical damage in the visual cortex is considered permanent, and there are no treatments that can restore lost vision. Interventions that do exist can be divided into restitution, compensation and substitution [15,16].

Restitution is the attempt to restore the absent visual field. These interventions include perceptual training—repetitive viewing and responding to simple visual stimuli, with a view to reducing the size of the lost field, or increasing contrast sensitivity where it has been reduced [17,18,19]. Restitution also includes the repeated stimulation of the impaired visual field, in an attempt to reactivate the corresponding visual areas of the brain [20]. While there have been some promising results from these studies, at present there is insufficient evidence to draw conclusions about their effectiveness [1]. As such, restitutive therapies do not provide an immediate solution for those people currently living with the effects of visual field loss.

Compensation interventions are targeted at training people to maximise the usefulness of their remaining vision. This includes training on making rapid, saccadic eye movements [21,22], visual search strategies [23,24,25,26,27,28,29], eye-movements for reading [24,29] and new strategies of making use of cues such as colour in everyday activities [30]. The focus of all of these is to change the strategies developed through a lifetime of unimpaired vision so that they can be optimised for that person’s new visual field.

Substitution interventions make use of optical devices or modifications of the environment to improve visual functioning. These include the use of optical prisms within spectacles that act to shift the visual field [25,26,27,31,32,33,34,35] and typoscopes to provide a guide to eye movements to facilitate reading [6,8,36].

Several companies already using virtual reality headsets to support people with low vision (e.g., Acesight [37], eSight [38], or IrisVision [39]) offer an assortment of enhancements through the manipulation of a digital feed such as magnification, contrast and edge enhancement, colour adjustment, and text reading. There have been attempts to use minification to expand the visual field [40,41,42,43]. These unfortunately have not to be useful or successful for people with visual field loss.

The primary reason why substitution via minification fails for people with visual field loss is due to disorientation. Minifying the field of view changes the visual direction and reduces visual resolution [44]. In contrast to people with low vision, those with visual field loss in many cases have normal acuity in their unimpaired visual field, and their needs differ from many other visual impairments, with a common priority being safe navigation. Unfortunately, virtual reality technology is still developing and the VR pass through camera will often experience a lag, resulting in a video feed that is not truly live, which is also disorienting.

Finally, this is further exacerbated by the minified field of view, where everything is further than appears in the headset. This makes mobility unsafe, further increasing the risk of falls or injury.

### 1.2. Augmented Reality and the Future

Augmented reality technology provides a valuable opportunity to extend current substitutive approaches. Head mounted augmented reality displays (specifically smart glasses) provide two critical components that make them a promising technology for developing substitutive interventions. The first is a head-mounted camera that can capture the visual field from the user’s point of view. The second is a head-up display, that allows visual information to be overlaid onto the natural field of view. Augmented reality can thus be used to provide a similar substitution as is achieved using optical prisms [31,32], namely shifting a portion of the visual field to another location so that is can be seen. However, it is important to first establish how effective overlaying part of the visual field with a minified view is at increasing awareness of the field of view for the wearer.

### 1.3. The Current Experiment

In this study, we simulate scotomatous vision loss for normally-sighted young-adult participants and include a minified picture-in-picture overlay of the scene. This differs from a global minification in that the direct, undistorted view of the scene is retained, and supplemented with an additional, minified wide-field projection onto a virtual screen. We tested the efficacy of this approach with a control population using an artificial, simulated scotoma in which the view of a large region of the visual field, on the left or right of centre, was obscured.

Performance was tested using a line bisection task, in which a line is presented on the screen and the observer indicates its perceived midpoint by moving a mouse cursor. This was chosen as a simple and well-established task used in understanding the effects of visual field loss in stroke [45,46]. Furthermore, control participants with a simulated scotoma have been shown to produce the same pattern of responses as participants with a visual impairment [47]. This provides an opportunity to initially test the concept prior to recruiting a patient population. The line bisection task was used as a measure of observers’ spatial awareness. It is important to note that while line bisection tasks are often used to evaluate bisection biases due to real or simulated visual field defects this paper is concerned only with differences in performance between our three conditions. We acknowledge that the findings of performance on line bisection task are limited as to how far it generalises to real world tasks and note that we plan more practical tasks (such as visual search tasks) in follow up studies.

There were three conditions. In the baseline, there was no artificial scotoma and no augmentation. In the artificial scotoma condition, we simulated a scotoma by masking a region of the visual field, which lay over a portion of the line to be bisected. In the augmentation condition, a windowed view was provided in addition to the scotoma, so that the line (and a spatial reference) could be seen in full within the window. We predicted that, if our design was successful, then performance would be substantially impaired in the artificial scotoma condition, and return towards baseline performance in the augmented condition.

## 2. Methodology

### 2.1. Participants

33 participants (20 females, mean (*std*) age 23.8 (*5.6*)) completed the experiment. All had normal or corrected to normal vision. All work was carried out in accordance with the Code of Ethics of the World Medical Association (Declaration of Helsinki). The study procedures were approved by the University of Essex Ethics Committee (Application No. JMA1901). All participants gave informed written consent and received a cash payment for their participation.

### 2.2. Apparatus and Stimuli

All stimuli were created using MATLAB and the Psychophysics [48,49,50] and Eyelink [51] Toolboxes. Stimuli were presented on a 67 cm × 28 cm monitor with a resolution of 2650 × 1080 px, and a 60 Hz refresh rate, with a viewing distance of 45 cm. Each pixel was 0.263 mm and subtended 2 arc min. Eye-position was monitored using an EyeLink 1000 eye-tracker (SR-Research Ltd., Mississauga, ON, Canada) where the sampling rate was 1000 Hz and recorded from the right eye.

Stimuli comprised six components which varied between three conditions; all stimuli were presented on a mid grey background that filled the screen, with the following components:

i. a white fixation cross: this was shifted 10${}^{\circ }$ to the left or right of the centre of the screen, depending on group. The fixation cross was 2${}^{\circ }\times 2^{\circ }$ and the fixation window for the eye tracker was 3.3${}^{\circ }\times 3.3^{\circ }$. The large fixation window allowed participants to remain fixated and view the augmentation window (when presented) without losing fixation. Eye tracking was used for fixation monitoring only, stimuli were only present when fixation was met. When fixation was lost, the stimulus disappeared.
ii. a black target line that was 0.3${}^{\circ }$ thick and one of 22 lengths, categorised as short (6.7${}^{\circ }$, 8.0${}^{\circ }$, 9.3${}^{\circ }$, 10.7${}^{\circ }$, 12.0${}^{\circ }$, 13.3${}^{\circ }$, 14.7${}^{\circ }$, 16.0${}^{\circ }$), medium (17.3${}^{\circ }$, 18.7${}^{\circ }$, 20.0${}^{\circ }$, 21.3${}^{\circ }$, 22.7${}^{\circ }$, 24.0${}^{\circ }$, 25.3${}^{\circ }$) or long (26.7${}^{\circ }$, 28.0${}^{\circ }$, 29.3${}^{\circ }$, 30.7${}^{\circ }$, 32.0${}^{\circ }$, 33.3${}^{\circ }$, 34.7${}^{\circ }$). Each participant was presented with only 16 of the 22 line lengths, which was randomised between participants, but held constant for all conditions for each individual. This provided us with three broad categories of line length (short, medium and long) whilst still allowing variation within and between participants [52].
iii. a black reference line with a fixed length of 42.8${}^{\circ }$ and 0.3${}^{\circ }$ thick, that was presented 6${}^{\circ }$ below the target line and centred on the screen. The reference line played an important role in the augmentation condition, but did not directly form a part of the bisection task.

The scotoma condition contained all the above elements, as well as:

iv. a simulated scotoma consisting of an overlaying mid grey oval with a horizontal radius of 13${}^{\circ }$ and a vertical radius of 6.67${}^{\circ }$, with its edges smoothed by a Gaussian luminance profile with a standard deviation of 18.1 arc minutes. The scotoma was placed $\pm 20.1^{\circ }$ left or right of the fixation cross, depending on the group, but not gaze contingent.

The augmentation condition contained all the above elements, including the simulated scotoma, plus:

v. the support window that was created with the same aspect ratio as the screen. The support window was 512 × 216 px ($17.1^{\circ }\times 7.2^{\circ }$), and the base of the window was positioned 1${}^{\circ }$ above the fixation cross. A scaled down copy of the whole of the screen, with the exception of the cursor, was presented in the support window. The fixation cross, target line, and reference line were all replicated in the support window.

### 2.3. Procedure

Prior to beginning the experiment observers were randomly allocated to a left-blind or right-blind group, briefed verbally and received a short demo containing one trial for each condition. The eye tracker was calibrated using a 9 point calibration at the start of each condition, and a self-calibration after each trial.

#### 2.3.1. Baseline Condition

**Figure 1:**
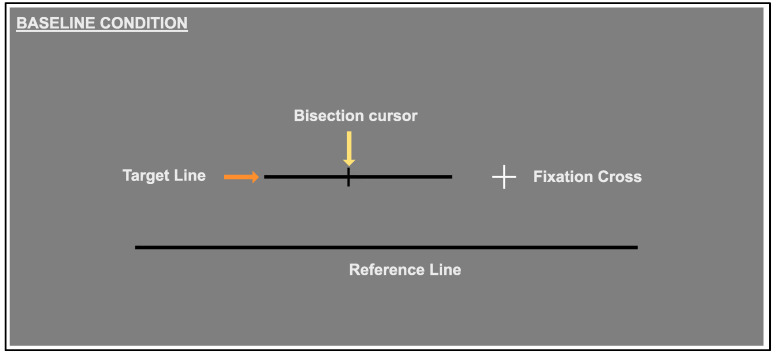
Baseline Condition: Target lines (of varying lengths) appeared at a randomised distance from the fixation cross, which was to the left of centre for the right-blind, and right of centre for left-blind conditions. A reference line was presented below the target.

All participants completed the baseline condition first, with the following two conditions randomised prior to starting. On each trial, a fixation cross was presented to the left of centre for the right-blind, and to the right of centre for the left-blind conditions. A target line (of varying length) appeared at a randomised distance and was always presented on the same side (either left or right) for an observer. The reference line was presented below the target (see Figure 1). This played an important role in providing a visual context in the augmentation condition, but did not directly form a part of the bisection task. Observers were required to keep their eyes fixated on the fixation cross. If participants moved their fixation further than 100 arc minutes from the centre of the fixation cross, the target, reference and cursor all disappeared from view and they were not able to respond. The stimulus reappeared once fixation returned to the cross. The bisection cursor was placed at a random position (on the horizontal axis) at the start of each trial. The participant used a mouse to move this along the horizontal axis until it appeared to bisect the target line.

#### 2.3.2. Scotoma Condition

**Figure 2:**
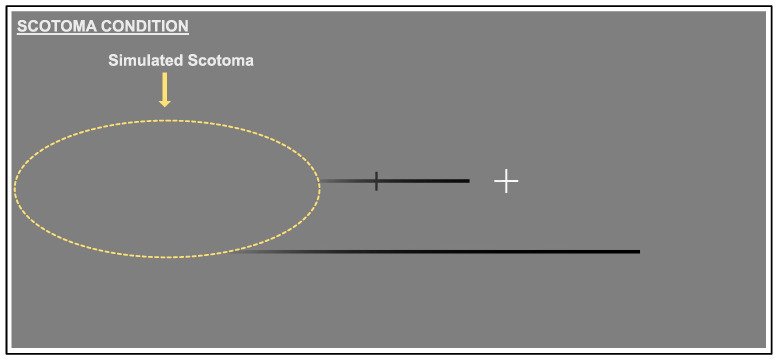
Scotoma Condition: The same configuration as the baseline condition, but a grey oval (smoothed by a Gaussian luminance profile) was overlayed so as to obscure a proportion of the target line. The cursor was not visible when within the scotoma.

The scotoma condition (see Figure 2) was identical to the baseline condition, however a simulated scotoma overlayed a portion of the screen. Nothing beneath the scotoma was visible.

#### 2.3.3. Support Window Condition

**Figure 3:**
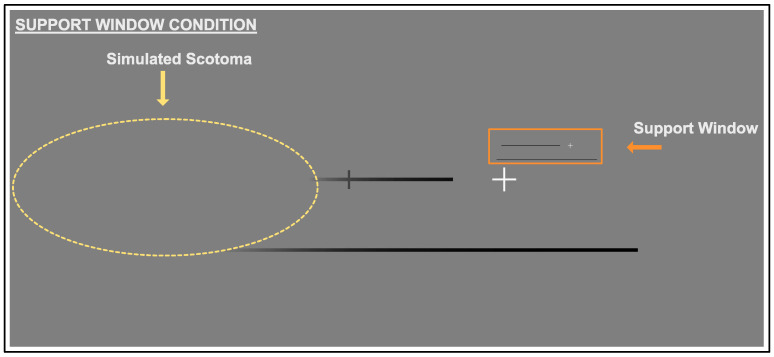
Augmentation Condition: In this final condition, the artificial scotoma was still present, but also included an image of the target and references lines was presented above fixation.

In the augmentation condition (see Figure 3), the artificial scotoma was still present, in addition a miniature image of the target and reference lines was presented above fixation. The cursor was not present in this augmentation window, to ensure that the task was completed in the stimulus space, rather than just in the window. The visibility of the reference in both the original space and the window allowed it to be used as a reference- within the window the observer should be able to see the length and location of the target (relative to the reference)—even if it was obscured by the artificial scotoma.

## 3. Results

Participants were required to use the mouse to click the point on the line that marked the midpoint. Responses were used to calculate the mean deviation from the midpoint in degrees of visual angle for each participant, condition (baseline, scotoma and window) and line-length (short, medium and long). Mean deviation was calculated in two formats, firstly the absolute mean deviation from the midpoint, and secondly the signed mean deviation from the midpoint.

### 3.1. Absolute Deviation

**Figure 4:**
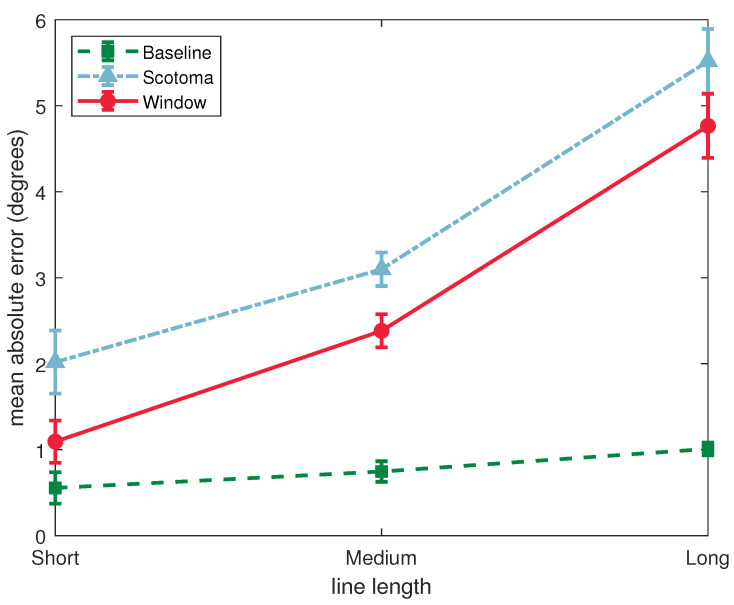
Results show absolute deviation from midpoint, where all responses report an underestimation (on average).

A 3 × 3 (condition by line-length) repeated measures ANOVA found statistically significant main effects for condition (*F*(2, 64) = 129.8, *p <* 0.001, $\eta_{p}^{2}$ = 0.802) and line-length (*F*(2, 64) = 47.2, *p <* 0.001, $\eta_{p}^{2}$ = 0.596). There was a significant interaction between condition and line-length (*F*(4, 128) = 19.4, *p* < 0.001, $\eta_{p}^{2}$ = 0.378) (see Figure 4).

Our primary focus was to determine whether (1) the artificial scotoma successfully led to an impairment of performance in the bisecton task and (2) whether the window improved performance relative to the scotoma condition. These comparisons were addressed using a Tukey corrected post hoc test for the effect of condition. Errors were significantly higher in the scotoma condition than in the baseline condition (*t*(32) = −18.73, *p <* 0.001). Errors were also significantly lower in the support window condition than in the scotoma condition (*t*(32) = 4.05, *p <* 0.001).

The significant interaction between the effects of condition and line-length suggest that the effects of the scotoma, and/or the support window, may have differed for different lengths of lines. This was followed up with one-way repeated measures ANOVAs to assess the effect of condition separately for each line length. This allowed us to determine whether the effects of condition were present across the range of stimuli used.

For the short stimuli, there was a significant effect of condition (*F*(2, 64) = 11.6, *p <* 0.001, $\eta_{p}^{2}$ = 0.266). Tukey corrected pairwise comparisons showed that errors were higher in the scotoma condition than than the baseline (*t*(32) = −4.17, *p <* 0.001), and lower with the support window than just the scotoma (*t*(32) = 2.52, *p =* 0.043).

For the medium stimuli, there was also a significant effect of condition (*F*(2, 64) = 56.3, *p <* 0.001, $\eta_{p}^{2}$ = 0.638). Tukey corrected pairwise comparisons showed that errors were higher in the scotoma condition than than the baseline (*t*(32) = −9.98, *p <* 0.001), and lower with the support window than just the scotoma (*t*(32) = 2.78, *p =* 0.024).

For the long stimuli, there was again a significant effect of condition (*F*(2, 64) = 93.4, *p <* 0.001, $\eta_{p}^{2}$ = 0.745). Tukey corrected pairwise comparisons showed that errors were higher in the scotoma condition than than the baseline (*t*(32) = −11.73, *p <* 0.001). In this case however there was no significant difference in the accuracy of bisection between the support window and scotoma conditions (*t*(32) = 2.43, *p =* 0.53).

This analysis shows that, while the effect of the artificial scotoma was to impair performance on the line bisection task for all line lengths, the beneficial effects of the support window were stronger for the shorter stimuli.

### 3.2. Signed Deviation

An analysis of the signed deviations was performed to determine whether errors represented a bias in the judgement of the location of the midpoint, or simply imprecision in the observers’ settings. For both the left and right conditions, a setting that was located further away from fixation than the actual midpoint of the line was coded as a positive error, and a setting that was made too close to fixation was coded as a negative error. This means that when the target line and scotoma were on the left, a positive error was in the leftward direction, and when they were on the right, a positive error was in the rightward direction.

**Figure 5:**
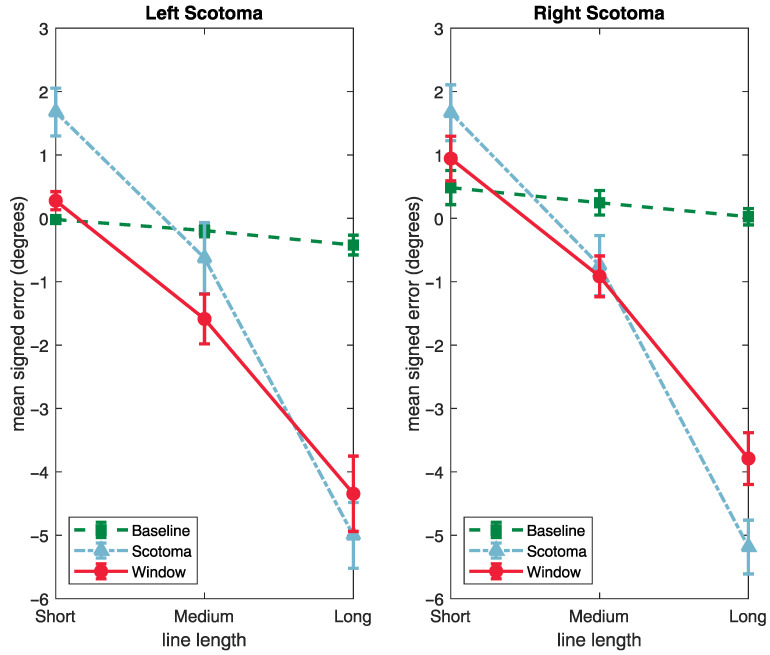
Negative errors are an underestimation where positive errors are an over estimation.

Signed errors are plotted in Figure 5, as a function of condition and line length, separately for the left and right conditions. These results show that, compared with the baseline condition, observers with both a left- and right-side artificial scotoma tended to underestimate the midpoint. This error was reduced by the presence of the support window.

These data were analysed using an ANOVA with condition (baseline, scotoma and window) and line-length (short, medium and long) as repeated measures factors, and the side of artificial scotoma (left or right) as a between subjects factor.

These results showed significant effects of condition (*F*(2, 62) = 10.7, *p <* 0.001, $\eta_{p}^{2}$ = 0.256) and line-length (*F*(2, 62) = 272.5, *p <* 0.001, $\eta_{p}^{2}$ = 0.898). There was a significant interaction between condition and line-length (*F*(4, 124) = 88.73, *p* < 0.001, $\eta_{p}^{2}$ = 0.808) (see Figure 5). There was no significant effect of the side of the artificial scotoma, *F*(1, 31) = 0.401, *p =* 0.531, $\eta_{p}^{2}$ = 0.013), nor any interactions between this and the repeated measures factors.

Left and right conditions were merged and analysed for the absolute deviation from the mid point.

Tukey corrected post hoc tests showed that the signed error was larger in the artificial scotoma condition than the baseline (*t*(31) = −19.29, *p <* 0.001), and smaller in the window condition than the artificial scotoma (*t*(31) = 4.00, *p <* 0.001). This shows that the support window was successful in reducing the error that resulted from the presence of the scotoma.

The significant interaction between condition and line-length was again followed up by performing separate analysis for each line length.

For the short stimuli, there was a significant effect of condition (*F*(2, 62) = 14.63, *p <* 0.001, $\eta_{p}^{2}$ = 0.321), but no effect of side (*F*(1, 31) = 0.013, *p =* 0.910, $\eta_{p}^{2}$ = 0.000), or interaction. A Tukey’s corrected post hoc test showed that error were larger in the presence of the artificial scotoma condition than the baseline (*t*(31) = −4.68, *p <* 0.001), and that this reduced in the presence of the support window (*t*(31) = 2.55, *p =* 0.041).

For the midlength stimuli, there was a significant effect of condition (*F*(2, 62) = 85.75, *p <* 0.001, $\eta_{p}^{2}$ = 0.734), but no effect of side (*F*(1, 31) = 2.02, *p =* 0.165, $\eta_{p}^{2}$ = 0.061), or interaction. A Tukey’s corrected post hoc test showed that error were larger in the presence of the artificial scotoma condition than the baseline (*t*(31) = −13.56, *p <* 0.001), and that this reduced in the presence of the support window (*t*(31) = 2.74, *p =* 0.027).

For the long stimuli, there was a significant effect of condition (*F*(2, 62) = 155.36, *p <* 0.001, $\eta_{p}^{2}$ = 0.835), but no effect of side (*F*(1, 31) = 0.009, *p =* 0.924, $\eta_{p}^{2}$ = 0.000), or interaction. A Tukey’s corrected post hoc test showed that error were larger in the presence of the artificial scotoma condition than the baseline (*t*(31) = −16.27, *p <* 0.001), but that this was not reduced in the presence of the support window (*t*(31) = 2.39, *p =* 0.058).

**Figure 6:**
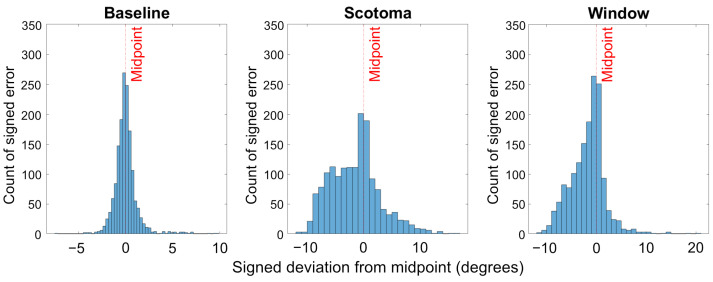
Histogram illustrating the response counts across all participants, collapsed across line lengths. Accuracy and precision improves in the window condition (by becoming more normally distributed compared to the Scotoma condition.

These results reflect the findings of the analysis of absolute errors, that the effect of the support window tends to be most evident for the shorter stimuli, and to reduce as line length is increased. Overall the inclusion of the window improves accuracy and precision in the line bisection task as demonstrated by the data becoming more normally distributed compared to the Scotoma condition (see Figure 6). Finally, this analysis shows that, for our line bisection tasks, observers’ response to the presence of the artificial scotoma was to indicate a location that was shifted into the periphery relative to the actual centre of the target line.

## 4. Discussion

The current study assessed whether a substitutive augmentation, in the form of a scaled image of the visual field presented in a window within the unimpaired visual field, can help to improve visual awareness. We tested this idea using a simulated scotoma, and a line bisection task as a measure of observers’ spatial awareness. As previously commented this paper is concerned only with differences in performance between our three conditions, rather than any pre-existing debates on biases in line bisection per se. The line bisection task was well suited to this goal since performance on the task is severely impaired by the artificial scotoma, but improved performance should be possible if observers are able to make appropriate use of the information provided in the support window. The negative errors in the left scotoma baseline condition, and positive error in the right scotoma baseline condition, both reflect a mean setting to the right of the actual midpoint. This bias is thus not consistent with the pseudoneglect found in line-bisection tasks under free viewing [53].

Our results show that, in comparison with our baseline condition, overall errors in identifying the centre of the target line were larger when the simulated scotoma was present.

In this condition, observers were unable to see the true length of the line, so it was not possible to accurately identify the location of its midpoint. In this case, a number of strategies were available. For example, observers could bisect the visible portion of the line, which would have led to a severe underestimation of the midpoint location. Alternatively, they may have assumed that the line extended throughout the unseen area obscured by the simulated scotoma, which would have led to an overestimation. Our results show an underestimation of the distance of the midpoint from fixation, indicating a conservative approach to extrapolating how far the target line might extend into the scotoma.

This bias was reduced in the window condition, in which performance was overall more accurate. This result shows that a substitutive augmentation of this type can be effective in improving performance at spatial tasks in the presence of a (simulated) scotoma.

In our implementation, we chose to substitute not just the region obscured by the simulated scotoma, but a much wider portion of the visual field. This provided a visual context that could be used to integrate the information contained in the support window with that visible in the unoccluded visual field. In our case, this allowed observers to judge the length and location of the target line relative to the extended reference line, allowing them to make a more informed estimate of its midpoint.

Another important feature of our design is that we did not present the cursor that was used to bisect the line within the support window. This means that observers were required to make use of the displayed stimulus itself, rather than its replication in the support window, in order to complete the task. This distinction reflects our intention that the support window represents an augmentation, providing additional visual information to enhance spatial awareness, rather than a miniaturisation of the view of the actual stimulus.

In our task, observers were required to maintain fixation, and the stimulus was presented to the left or right of fixation. In unrestricted viewing, we would expect observers to make many eye-movements across the stimulus, and that these would differ in the scotoma and non-scotoma conditions as observers sampled the available information. This restriction means that our aim was not to replicate the visual behaviour that might be expected in natural viewing. Rather, the combination of a single fixation location and the artificial scotoma was used to create a situation in which the information available across the visual field was severely limited. Our goal was to address the extent to which people were able to use the information provided in the support window to overcome this limitation and improve performance.

These results are a first step in demonstrating that this type of augmentation is beneficial for improving performance on a simple visual task. An important next step will be to demonstrate similar improvements in important real-world visual tasks, such as navigation and obstacle avoidance. It is also important that the approach is tested with observers with actual visual field loss, building on these results for a simulated scotoma. Research with this population should then assess the extent to which this augmentation leads to improvements in visual function and quality of life. Based on these preliminary findings we predict that individuals with visual field loss would benefit from this augmentation particularly for near objects. This might be particularly useful for hazard detection.

The image processing capability of Augmented Reality potentially provides much greater flexibility in how this information is captured and displayed. For example, the size and location of the field captured, and the size and location of the presentation of this information to the user in the head-up display, can all be manipulated independently without obscuring the normal visual field.

## Data Availability

Data will be uploaded to OSF once paper is accepted.

## Abbreviations

The following abbreviations are used in this manuscript:

AR	Augmented Reality
VR	Virtual Reality
VFL	Visual Field Loss
